# Drag, but not buoyancy, affects swim speed in captive Steller sea lions

**DOI:** 10.1242/bio.20146130

**Published:** 2014-04-25

**Authors:** Ippei Suzuki, Katsufumi Sato, Andreas Fahlman, Yasuhiko Naito, Nobuyuki Miyazaki, Andrew W. Trites

**Affiliations:** 1 Atmosphere and Ocean Research Institute, The University of Tokyo, 5-1-5 Kashiwanoha, Kashiwa, Chiba 277-8564, Japan; 2 Department of Natural Environmental Study, Graduate School of Frontier Sciences, The University of Tokyo, 5-1-5 Kashiwanoha, Kashiwa, Chiba 277-8564, Japan; 3 Department of Life Science, Texas A&M University-Corpus Christi, 6300 Ocean Drive, Corpus Christi, TX 78412, USA; 4 Department of Zoology and Marine Mammal Research Unit, Fisheries Center, University of British Columbia, 2204 Main Mall, Vancouver, BC V6T 1Z4, Canada; 5 National Institute of Polar Research, 10-3 Midoricho, Tachikawa, Tokyo 190-8518, Japan

**Keywords:** Cost of transport, Optimal swim speed, Diving, *Eumetopias jubatus*

## Abstract

Swimming at an optimal speed is critical for breath-hold divers seeking to maximize the time they can spend foraging underwater. Theoretical studies have predicted that the optimal swim speed for an animal while transiting to and from depth is independent of buoyancy, but is dependent on drag and metabolic rate. However, this prediction has never been experimentally tested. Our study assessed the effects of buoyancy and drag on the swim speed of three captive Steller sea lions (*Eumetopias jubatus*) that made 186 dives. Our study animals were trained to dive to feed at fixed depths (10–50 m) under artificially controlled buoyancy and drag conditions. Buoyancy and drag were manipulated using a pair of polyvinyl chloride (PVC) tubes attached to harnesses worn by the sea lions, and buoyancy conditions were designed to fall within the natural range of wild animals (∼12–26% subcutaneous fat). Drag conditions were changed with and without the PVC tubes, and swim speeds were recorded and compared during descent and ascent phases using an accelerometer attached to the harnesses. Generalized linear mixed-effect models with the animal as the random variable and five explanatory variables (body mass, buoyancy, dive depth, dive phase, and drag) showed that swim speed was best predicted by two variables, drag and dive phase (AIC = −139). Consistent with a previous theoretical prediction, the results of our study suggest that the optimal swim speed of Steller sea lions is a function of drag, and is independent of dive depth and buoyancy.

## INTRODUCTION

Breath-hold divers have morphologically and physiologically evolved to live in aquatic environments. A streamlined body shape is one of the most distinct adaptations essential to reduce drag in a high viscosity environment (Fish, 1996; Fish et al., 2008). In addition to reducing drag, breath-hold divers have morphological traits that allow efficient propulsive forces with paddle- or hydrofoil-like appendages (Berta et al., 2006). These morphological adaptations allow the animals to efficiently swim and dive, but do not address the limiting effects of a restricted oxygen supply on time spent underwater.

The physiological adaptations for economical usage of oxygen are generally known as the dive response, which includes a decrease in heart rate and a restricted peripheral blood flow to conserve the available O_2_ for the heart and brain (Hill et al., 1987; Kooyman, 1989; Butler and Jones, 1997; Kooyman and Ponganis, 1998). A decrease in peripheral body temperature also plays an important role in reducing metabolic costs while underwater (Handrich et al., 1997). In addition, various marine vertebrates augment these physiological traits using a range of behavioral responses that are believed to extend time spent underwater (Kramer, 1988).

Optimal foraging theory suggests that breath-hold divers should maximize the time that they can stay at depths where their prey occur. This in turn suggests that an animal should minimize the energy it expends transiting to and from depth. A number of diving data sets (especially from wild benthos-feeders) have shown marine mammals maintain relatively constant speeds during both the descent and ascent phases compared with the bottom phases of their dives (Boyd et al., 1995; Crocker et al., 2001; Watanabe et al., 2006). These constant swimming speeds between the surface and depth-of-feeding suggests they are optimum speeds and that they are likely a function of physical laws. However, swim speeds while feeding in the bottom phase tend to vary and are less predictable due to differences in feeding behaviors associated with searching, chasing, grabbing, and handling (Wilson et al., 2002).

The energetic cost of animal locomotion (running, swimming, and flying) has been discussed since the early 1970s (Tucker, 1970; Schmidt-Nielsen, 1972), and has been termed the cost of transport (COT) – the metabolic cost required to transport the animal's mass over a unit of distance (Prange and Schmidt-Nielsen, 1970). Comprehensive studies have revealed that higher basal metabolic rates imply higher optimal speeds that minimize COT to compensate for increased time-dependent metabolic costs with speed-dependent mechanical costs (Videler and Nolet, 1990; Williams, 1999; Alexander, 1999; Sato et al., 2010). Models that describe the energetic cost of breath-hold diving generally assume that COT is minimized during descent and ascent phases (Thompson et al., 1993; Gallon et al., 2007).

A recent study (Sato et al., 2010) developed a theoretical model to calculate the optimal swim speed with diving data sets from seven species of geometrically similar penguins. Sato et al. calculated the cost of transport of the descending penguins as a function of both metabolic and mechanical costs. They determined metabolic costs of diving to given depths at a constant swim speed and pitch angle; and they estimated mechanical costs from calculated thrust force using drag, buoyancy, and pitch angle. Sato et al. found that steeper dive angles resulted in a lower COT because shorter descent times reduced metabolic costs. They also found that maintaining a constant swim speed was the optimal thing for penguins to do for all pitch angles (Sato et al., 2010).

The optimal swim speed can be calculated using the following equation (Sato et al., 2010):
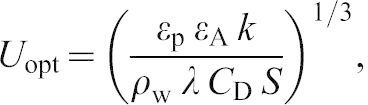
(1)where *ε*_p_ is the propeller efficiency, which is the translation ratio of muscle work to propulsive work; *ε*_A_ is the aerobic efficiency, which is the conversion ratio of chemical energy to kinetic energy (muscle work); *k* is the basal metabolic rate (J s^−1^); *ρ*_w_ is the density of the sea water ( = 1,027 kg m^−3^); *λ* is the ratio of the drag of an active swimmer to that of a passive object; *C*_D_ is the drag coefficient of gliding animals based on the total wetted surface area; and *S* is the total wetted surface area (m^2^). This predictive equation suggests that optimal swim speed is proportional to one-third of the power of the basal metabolic rate divided by the drag, and is independent of buoyancy, pitch angle, and dive depth.

According to the theoretical prediction, COT varies with buoyancy, whereas buoyancy does not influence the optimal swim speed. However, buoyancy is one of the most variable factors associated with the body condition of wild animals – as seen in pinnipeds where blubber and total body lipid content can vary by at least 10% of total body mass (Webb et al., 1998; Pitcher et al., 2000). Changes in body density have also been shown to significantly affect descent rates in buoyancy-manipulated northern elephant seals (Webb et al., 1998), as well as the stroke patterns in artificially weighted Baikal seals (Watanabe et al., 2006) and buoyancy-manipulated northern elephant seals (Aoki et al., 2011). However, in other species such as Steller sea lions, artificially changing the buoyancy within a range of naturally occurring differences in body composition observed in wild individuals (12–26%) (Pitcher et al., 2000) did not affect the metabolic cost of foraging (Fahlman et al., 2008a). These discoveries point to the need to experimentally identify the factors that determine optimal swim speed in pinnipeds and other breath-hold diving animals.

We tested the effects of buoyancy, dive depth, and drag conditions on the swim speeds of trained Steller sea lions that wore harnesses equipped with accelerometers (to record swim speed) and a pair of buoyancy tubes (to manipulate buoyancy and drag conditions). We sought to experimentally determine whether the theoretical optimal swim speed is dependent on drag and independent of buoyancy and diving depth. This represents the first experimental attempt to confirm this theoretical prediction by manipulating the conditions thought to influence optimal swim speeds. Our findings have bearing on the reliability of the model (Eqn 1) to estimate the COT of breath-hold divers using swim speed.

## MATERIALS AND METHODS

All experiments were conducted under permits issued by the Animal Care Committees of the University of British Columbia and the Vancouver Aquarium.

### Animals

Experiments were conducted in July and September 2007 using three female Steller sea lions *Eumetopias jubatus* housed in a specially designed floating pen located in the Indian Arm (49° 19.917′ N, 122° 55.005′ W) in British Columbia, Canada. The sea lions freely chose to cooperate with all data collection and were never restrained during any of the experimental trials. The sea lions were 7 (F00BO) and 10 years old (F97HA and F97SI). The body mass (*M*_b_) of each sea lion was measured daily in the morning prior to the experiments and were averaged (± SD) over the experimental period (Table 1).

**Table 1.**
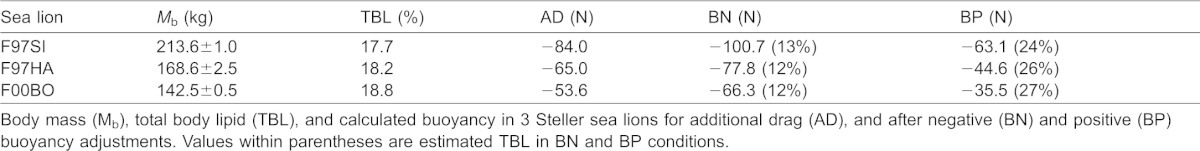
Eumetopias jubatus

### Buoyancy and drag adjustment

**Fig. 1.**
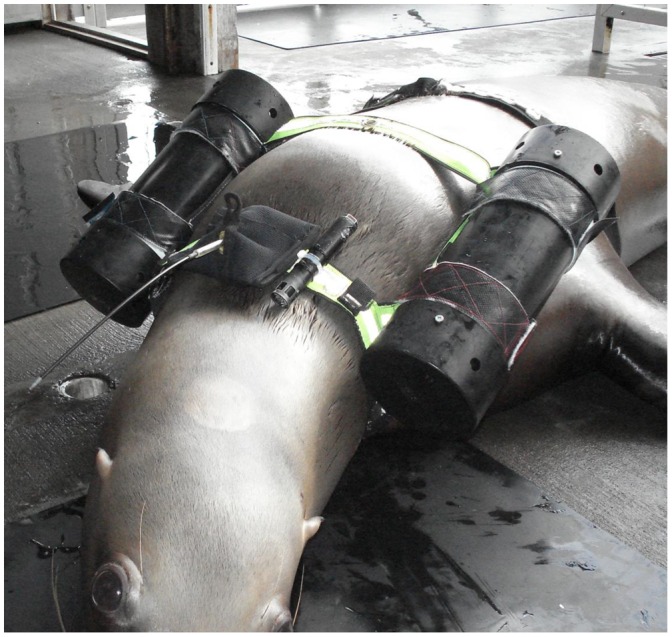
A Steller sea lion equipped with a harness containing a pair of side-mounted PVC tubes, a very high frequency transmitter with an antenna between the tubes, and a cylindrical accelerometer next to the transmitter.

Both buoyancy and drag conditions were manipulated using a pair of polyvinyl chloride (PVC) tubes (diameter: 12.5 cm; length: 35 cm) attached to a webbing body harness that was worn by each animal (Fig. 1). The method for estimating buoyancy and details of the tubes were previously described (Fahlman et al., 2008a). Briefly, total body water (TBW) was estimated using the deuterium dilution method and total body lipid (TBL) was calculated from the predictive equation reported for Antarctic fur seals (Arnould, 1995). The proportions of TBW and TBL were used to estimate total buoyancy with constant mass-specific buoyancy for lean tissue and adipose tissue as reported in a previous study (Webb et al., 1998). Buoyancy conditions were adjusted to fall within the maximal range of naturally occurring differences in body composition (12 to 26%) of wild adult female Steller sea lions (Pitcher et al., 2000).

Four types of trials were conducted to assess the effects of buoyancy and drag on swim speed. These included a control (C), additional drag (AD), negative buoyancy (BN), and positive buoyancy (BP). For C trials, the animal wore a harness equipped with a radio transmitter that allowed us to track the animal in case they left the experimental area. The harness increased the drag, but was a necessary safety precaution for the animal. It also allowed us to attach instruments to the animal. For the other trials, the animals always wore the harness with a radio transmitter and a pair of PVC tubes to manipulate drag and buoyancy conditions. Both front and rear ends of the PVC tubes were glued closed with PVC end caps to simulate the same drag in the three conditions with the tubes (AD, BN, and BP) for the frontal surface area, while holes (diameter: 5 cm) were drilled at the rear end to allow water to enter the tubes for the AD and BN trials.

For the AD trials, nothing was enclosed in the tubes as a control treatment for buoyancy manipulation, but it had more drag compared to the C trials due to the attachment of the tubes.

For the BN trials, the tubes were filled with a sheet of lead that represented the least buoyant condition (with an estimated body composition of 13%). The mass of the lead sheet was determined using the equation described in the previous studies (Webb et al., 1998; Fahlman et al., 2008a), and the lead sheets weighing 1.3 and 1.7 kg in the water (lighter for F97HA and F00BO, and heavier for F97SI) were enclosed in the tubes.

For the BP trials, the internal air volume was adjusted to simulate the most buoyant condition (at an estimated body composition of 24 to 27%), and an arbitrary amount of water filled the tubes before closing off the rear end to reduce extra buoyancy for the small animals as the size of PVC tubes was constant. The PVC tubes were designed not to change in volume due to ambient pressure, and therefore ensured that buoyancy conditions remained constant through each set of dives.

Estimated buoyancy and total body lipid for each condition are listed in Table 1. Drag effect was compared between the condition without buoyancy tubes (C trials) and the other three conditions with the tubes (AD, BN, and BP trials).

### Experimental procedure

**Fig. 2.**
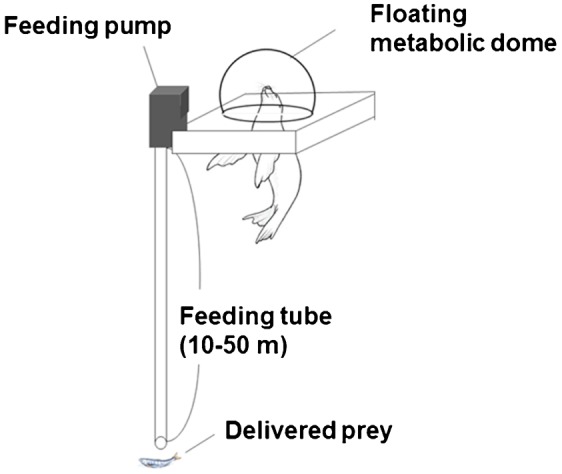
Schematic view of the experimental set up. The Steller sea lions were trained to breathe in the floating metabolic dome and to dive to fixed depths (10 to 50 m) where they could catch fish pumped down the feeding tube.

All experimental trials were performed in the morning, at least 16 h postprandial, and conducted at the trial area in the Indian Arm as previously described (Fahlman et al., 2008a; Fahlman et al., 2008b), in which animals started diving from a floating respirometry dome to an underwater feeding tube placed at a predetermined depth of up to 50 m (Fig. 2). Prior to each experimental trial, each animal was weighed (±0.5 kg) and fitted with the harness that held an accelerometer (see the following subsection) and a radio transmitter. Each animal was then transported to the trial area using a 22-ft boat and the buoyancy tubes were attached to the harness.

Before diving, the sea lion stayed in the dome to measure its metabolic rate at the surface using flow-through respirometry (Fahlman et al., 2008a). After a period of 6 to 8 min at the surface, each sea lion was instructed to dive to the end of the tube where pieces of defrosted Pacific herring *Clupea pallasii* (approximately 20 g per piece) were delivered at a constant rate (2–4 pieces per second). Herring was continuously delivered until the sea lion left and started to ascend, which was confirmed by a camera monitor placed at the bottom of the tube, to simulate their natural feeding behavior to maximize their time to stay in the bottom and assume the arrival of a prey patch.

### Estimation of swim speed

A cylindrical accelerometer (W2000-PD2GT: 21 mm in diameter, 117 mm in length, with 60-g air; Little Leonardo Ltd., Tokyo, Japan) was attached to the harness, and recorded depth at 1 Hz, two-axes of acceleration (longitudinal and dorso-ventral axis) at 32 Hz, and temperature at 1 Hz. The longitudinal acceleration was converted to the pitch angle of the diving animal by extracting gravitational acceleration (Sato et al., 2003).

Descent and ascent durations were extracted based on the depth and pitch angle. Descent duration started from the point when the animal began heading down to the point when it reached the fixed depth. Ascent duration was from the point when the animal started to head up to the point when it reached the surface.

**Fig. 3.**
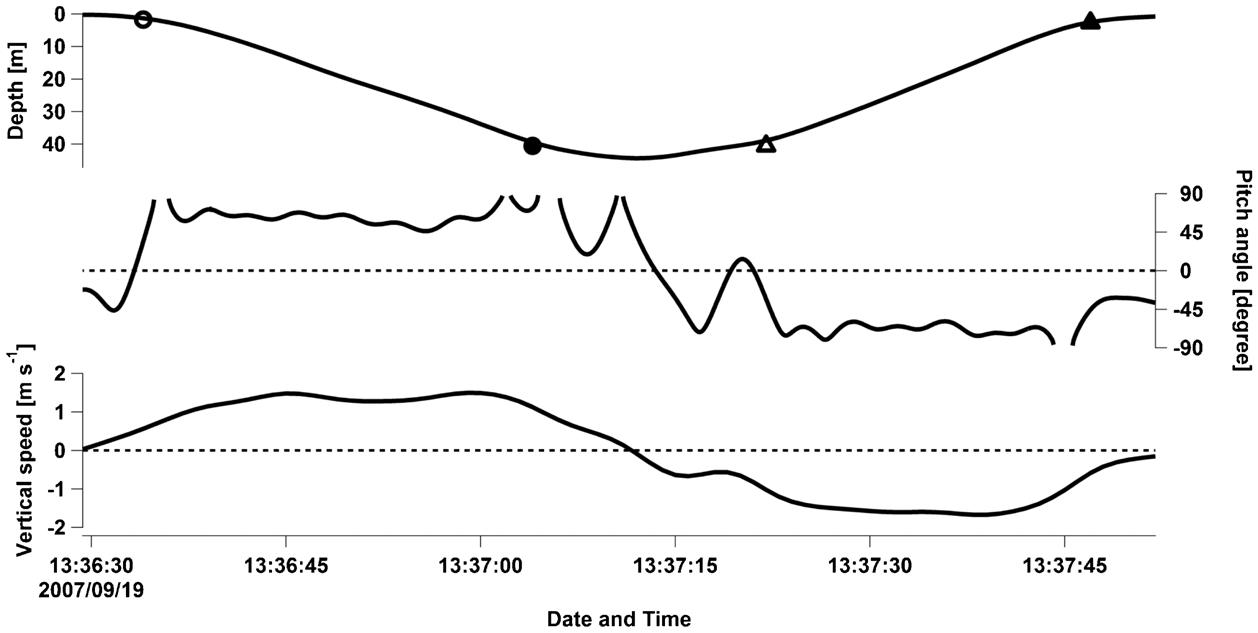
An example of a dive profile obtained from a 213-kg Steller sea lion (F97SI) showing the dive depth (top), pitch angle (middle), and vertical speed (bottom). Symbols in the depth data indicate the start of descent (open circle), the end of descent (bold circle), the start of ascent (open triangle), and the end of ascent (bold triangle). The definition for each point is described in the text. Broken lines in the pitch angle mean impossibility to convert the unit in degrees due to acceleration values exceeding 9.8 m s^−2^. Constant pitch angle and vertical speed during descent and ascent phases suggest that the animal is in a quasi-steady state during these phases.

Dive profiles showed that the pitch angle and vertical swim speed (obtained from the depth data as differences between each second) were almost constant during both descent and ascent phases (Fig. 3). The constant pitch angle and swim speed in the descent and ascent phases agreed with the reported dive profiles obtained from the wild breath-hold divers (Boyd et al., 1995; Crocker et al., 2001; Watanabe et al., 2006). We thus assumed that these phases were in a quasi-steady state during the period in which the optimal swim speed could be estimated from Eqn 1 (Sato et al., 2010). As the focus of our study was to examine the optimal swim speed and not vertical speed, all swim speeds (*U*) used in our analysis were calculated with the mean pitch angle using the following equation:
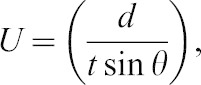
(2)where *d* is the fixed depth (m), *t* is either the descent or ascent duration (s), and *θ* is the mean absolute pitch angle (degree) during either the descent or ascent phase.

### Calculation of optimal swim speed

Optimal swim speed for each animal was estimated using Eqn 1 (Sato et al., 2010). The propeller efficiency (*ε*_p_) was set to 0.8 and aerobic efficiency (*ε*_A_) was set to 0.15 based on a captive experiment that used California sea lions (Feldkamp, 1987). The basal metabolic rate (*k*) in the Feldkamp study was considered to be the rate at which the animals consumed oxygen while breathing in the floating dome. We considered this to be the resting metabolic rate in the water (RMR_w_), which we subtracted from total metabolic power input to obtain the net power available for swimming.

Our study was conducted concurrently with another set of experiments that measured oxygen consumption rate for a series of dives (Fahlman et al., 2008a). This meant that the energetic cost of single dives were unavailable. Measured RMR_w_ (l O_2_ min^−1^) were therefore taken from table 2 of Fahlman et al. (Fahlman et al., 2008a) and converted to Joules using a conversion factor of 20.1 J per mL O_2_ (Schmidt-Nielsen, 1997).

Due to limited information, the ratio of the drag of an active swimmer to that of a passive object (*λ*) was set to 1 similar to a previous study (Miller et al., 2012). We set the drag coefficient (*C*_D_) to either 0.0025 or 0.0098, which were the respective minimum and the maximum values calculated from the deceleration rate observed in captive Steller sea lions (Stelle et al., 2000).

Total wetted surface area (*S*) was estimated using an empirical equation obtained from reported values for *S* and body mass (M_b_) (Stelle et al., 2000). Assuming geometrical similarity within Steller sea lions, S was considered as a function of a two-third power of M_b_ using:
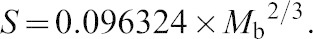
(3)To estimate the hydrodynamic effect of the PVC tubes on the animals, we calculated the drag coefficients for each animal with the PVC tubes. Our PVC tubes had an aspect ratio of 2.8:1 (length: diameter). According to a hydrodynamic study (Blevins, 1984), a cylindrical object with the aspect ratio of 3:1 has a *C*_D_ of 0.064. A drag coefficient including both an animal and a pair of PVC tubes can therefore be estimated with total drag area divided by total wetted surface area of the animal and the tubes. We calculated the wetted surface area of the animals from Eqn 3 and that of the tubes from the geometry of a cylinder. We also calculated the drag area of the tubes using a *C*_D_ of 0.071 (based on an object on a surface increasing the *C*_D_ of a cube by ∼10% (Blevins, 1984)), and summed the drag area of the animal and the tubes after multiplying each wetted surface area with each *C*_D_ (divided by the sum of surface area obtained from the animal and the tubes). Finally, we recalculated the drag coefficient of each animal with the PVC tubes using the initial drag coefficient of the animal ranging from 0.002 to 0.01, which covered the range of *C*_D_ measured in Steller sea lions (Stelle et al., 2000).

All parameters used in our study are listed in Table 2.

**Table 2.**
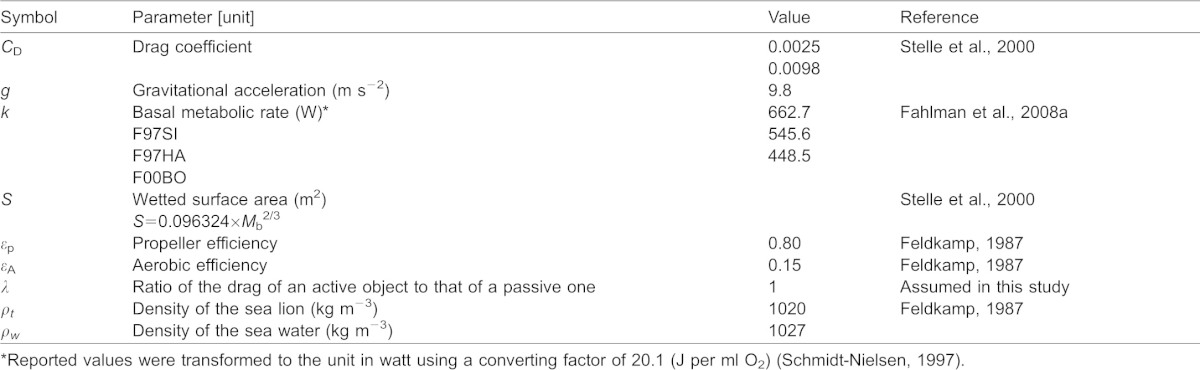
List of symbols and values used to estimate optimal swim speeds in *Eumetopias jubatus*

### Statistics

We used a generalized liner mixed-effect model (GLMM) to determine interactions among swim speed (dependent variable) and five explanatory variables (body mass, buoyancy, dive depth, drag, and dive phase: descent or ascent phase) with individual identification as a random effect (family  =  Gaussian; link  =  identity). A value from the Akaike's Information Criteria (AIC) for each model was used to select the most parsimonious model. The GLMM analysis was executed using the R 2.15.2 software using the *lmer* function in the R package *lme4* (D. Bates and M. Maechler, *lme4*: Linear mixed-effects models using S4 classes. R package version 0.999999-0, available from http://cran.r-project.org, 2009). All averaged data were associated with standard deviations.

## RESULTS

**Fig. 4.**
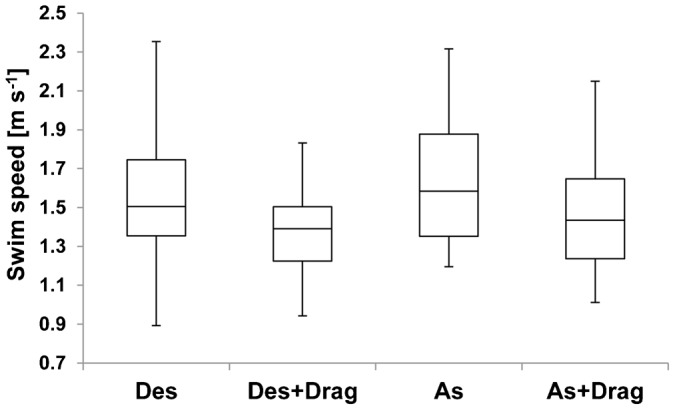
A box-and-whisker plot of measured swim speeds of 3 Steller sea lions (*Eumetopias jubatus*) during descent (Des) and ascent (As) phases, and while carrying drag tubes during descent (Des + Drag) and ascent (As + Drag) phases. GLMM analysis indicated that the swim speed was predicted by drag and dive phase with the minimum AIC value of −139 as shown in Table 5. The whiskers depict maximum and minimum values, and the boxes show the lower quartiles, the medians, and the upper quartiles.

**Fig. 5.**
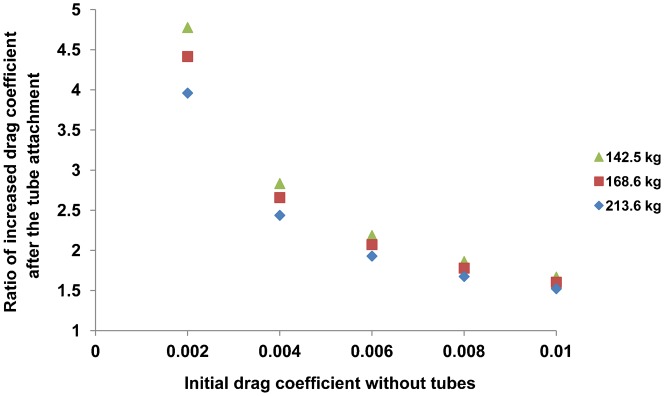
Increased ratio of drag coefficient after attaching a pair of PVC tubes against the initial drag coefficients (0.002 to 0.01) for female Steller sea lions of different sizes (142.5–213.6 kg).

The three sea lions performed a total of 186 individual dives (Table 3). Mean swim speeds without drag tubes were faster than those with the tubes in each phase (Fig. 4). Calculated optimal swim speeds ranged from 1.3 to 2.1 m s^−1^, and recorded mean swim speeds ranged from 1.4 to 2.0 m s^−1^ within the C trials (Table 4). According to the GLMM analysis, swim speed was predicted by two explanatory variables (drag and dive phase) with the minimum AIC value of −139 (Table 5). Swim speed during the ascent phase was faster than that during the descent phase (Fig. 4). Estimated drag coefficient after the tube attachment showed higher increase of hydrodynamic effect in smaller initial drag coefficient (Fig. 5).

**Table 3.**
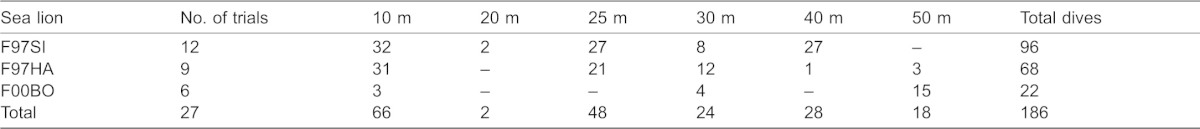
Total number of trials and number of dives to each depth for 3 Steller sea lions

**Table 4.**
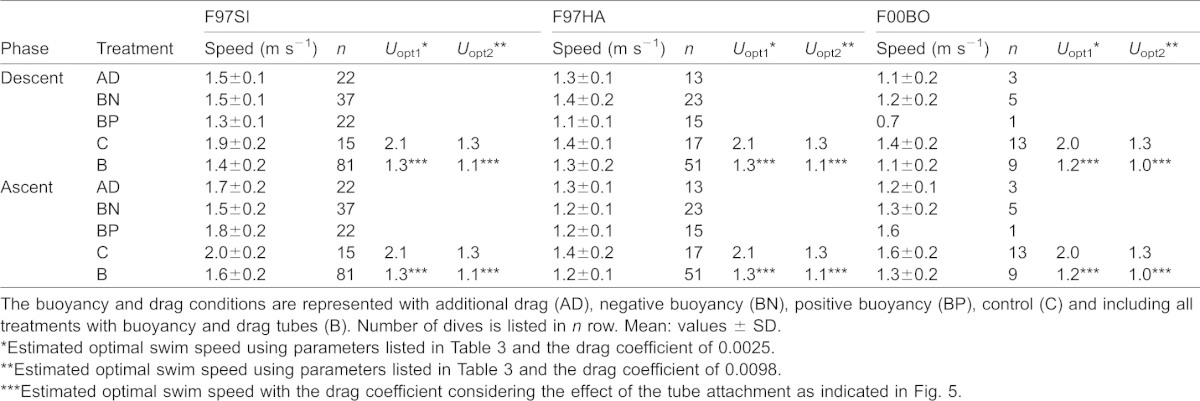
Swim speeds of *Eumetopias jubatus* in descent and ascent phases

**Table 5.**
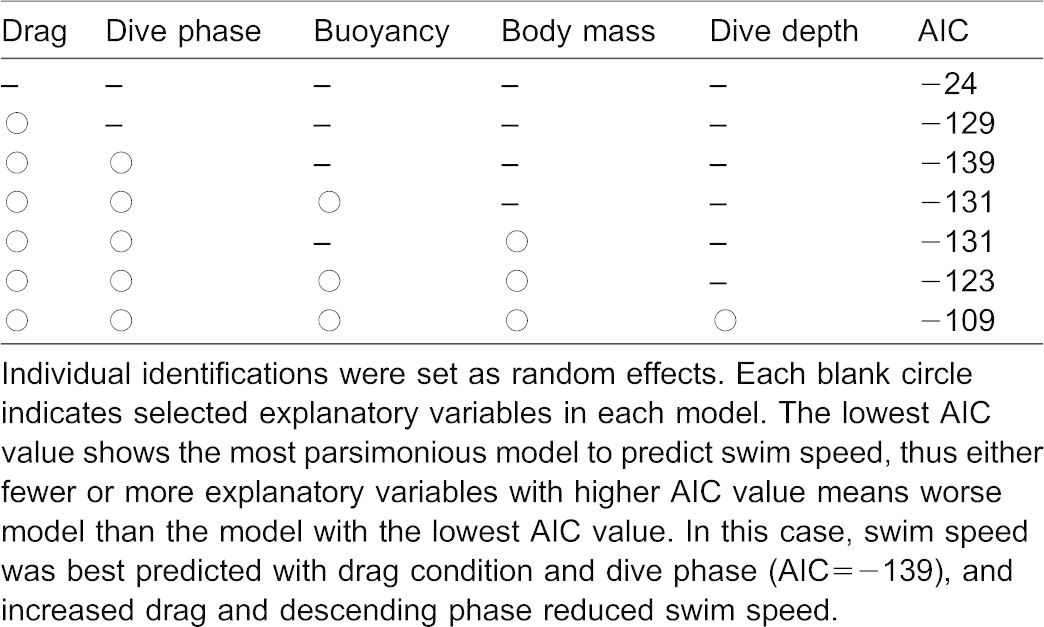
Results of AIC values from a generalized linear mixed-effect model (family  =  Gaussian, link  =  identity) to predict swim speed of Steller sea lions with five explanatory variables: drag, dive phase (descent/ascent), buoyancy, body mass and dive depth

## DISCUSSION

We measured the swim speed of three Steller sea lions under different buoyancy and drag conditions while undertaking dives to depths ranging from 10 to 50 m to determine the factors that affected swim speed. The GLMM results showed that swim speeds were affected by drag and the dive phase, and were not affected by buoyancy and dive depth (Table 5). This agrees with the prediction that optimal swim speed is a function of drag, and is independent of buoyancy and dive depth (Sato et al., 2010).

Our study provides the first experimental evidence that swim speeds of breath-hold divers are significantly decreased by artificially increased drag (Fig. 4). However, we had not expected to find a difference in swim speeds between the descent and ascent phases (Table 5). We suspect the difference reflects differences in the initial swim speeds of the animals at the start of each phase. For animals at the surface, the initial swim speeds during the descent phase were always close to zero as they began their dives, whereas the initial speeds in the ascent phase were seldom or never zero. Further experiments involving deeper dives (>50 m) may provide further insight into this apparent discrepancy, because initial swim speeds should have less effect on the average swim speed as the distance to the bottom is increased.

Buoyancy was not selected as an explanatory factor affecting the swim speed of our Steller sea lions, which agreed with the theoretical prediction of Sato et al. (Sato et al., 2010). This contradicts the results from northern elephant seals that showed a significant correlation between descent rate and the level of buoyancy adjustment (Webb et al., 1998). A modeling study involving southern elephant seals (Miller et al., 2012) also showed that slight changes from neutral buoyancy did not affect the optimal swim speed, whereas extreme changes altered swim speed in the buoyancy-aided direction. Considering the difference in naturally occurring ranges of body compositions between phocid and otariid seals (∼20–50% for phocids, and ∼15–30% for otariids) (Webb et al., 1998; Pitcher et al., 2000), phocid seals may be better equipped to compensate for variation in buoyancy while diving by changing their stroke patterns as reported in Baikal seals (Watanabe et al., 2006).

Steller sea lions tend to be relatively shallow divers compared to phocids and were diving in our experiment within the range of mean depths commonly observed in the wild (Merrick and Loughlin, 1997; Rehberg et al., 2009). Several studies involving phocid seals using artificially manipulated buoyancy conditions showed changes in terminal speed corresponding to the added body density in deep dives (>100 m) (Watanabe et al., 2006; Aoki et al., 2011; Miller et al., 2012), while no obvious buoyancy effects on swim speed in shallow dives (<50 m) were observed (Watanabe et al., 2006). Conducting further experiments using deeper dives (>100 m) may be one way to resolve this question about the effects of buoyancy on swim speed (given that buoyancy effects are sustained in deeper dives).

The calculated optimal swim speed (1.3–2.1 m s^−1^) was relatively close to the recorded swim speed (1.4–2.0 m s^−1^) in the C treatments (Table 4). The consistency in the minimum speed suggests that animals chose their optimal swim speed based on drag conditions. In doing our calculations, we used two values of *C*_D_ (0.0025 or 0.0098), which were the respective minimum and maximum *C*_D_ for the deceleration rate of captive Steller sea lions during gliding phases (Stelle et al., 2000). The calculated optimal swim speed using *C*_D_ of 0.0025 (*U*_opt1_; Table 4) was higher than the recorded speed, especially in smaller animals (F97HA and F00BO), whereas the calculated optimal swim speed using *C*_D_ of 0.0098 (*U*_opt2_; Table 4) fit better with the recorded speed for these animals.

We assumed that our C treatment was the closest to what a sea lion would naturally experience in terms of drag, but recognize that the harness must have increased *C*_D_ (which we did not measure). Calculating the optimal swim speed with a *C*_D_ of 0.0056 (the average *C*_D_ in the aquarium experiment from Stelle et al. (Stelle et al., 2000)) yields values of 1.6 m s^−1^ (F97HA) and 1.5 m s^−1^ (F00BO) for the two smaller animals. These are faster than the swim speeds we measured. However, recalculating optimum swim speeds with a *C*_D_ of 0.0098 (*U*_opt2_) yields values that are closer to the measured speeds. This agreement between the estimated optimal speeds (*U*_opt2_) and measured swim speeds in the smaller sea lions (F97HA and F00BO) using the maximum reported *C*_D_ suggests that increased drag decreased their optimal swim speeds.

The hydrodynamic effect of the PVC tubes on the animals also appears to explain the lower optimal swim speeds of the BN and BP trials. Our calculations of drag suggest that the sea lions experienced a 4 to 5 fold increase in drag while carrying the tubes (Fig. 5). In addition, we found a greater hydrodynamic effect on sea lions that had lower body masses because the lighter animals had smaller surface areas (Fig. 5). Recalculating the optimal swim speed using drag coefficients that accounted for the added effect of the tubes yields *U*_opt_ estimates of 1.3 (for F97SI), 1.2 (F97HA) and 1.2 m s^−1^ (F00BO) for the B trials, which are comparable to what we measured during the dive trials (Table 4). Similarly, applying a mean initial drag coefficient of 0.004 for the C trials (Williams and Kooyman, 1985; Feldkamp, 1987) results in estimates of optimum swim speeds of 1.8 (for F97SI), 1.8 (F97HA) and 1.7 m s^−1^ (F00BO) that are again in line with the speeds the animals attained when diving without the tubes (Table 4). These findings further support the conclusions that drag affects the optimal swim speeds of breath-hold divers.

Although it has been previously recognized that increased drag reduces speed when the propulsive force is constant, it has been difficult to confirm the effect of drag on swim speed using diving animals. Noren et al. compared the gliding speed of pregnant and non-pregnant bottlenose dolphins (*Tursiops truncatus*) and confirmed that pregnant individuals had a significantly higher drag and a slower swim speed than non-pregnant individuals (Noren et al., 2011). However, the effect of drag alone on swim speed has never been examined because pregnancy coincides with several physiological changes, including those involving the cardiovascular, hematologic, metabolic, and respiratory systems (Elsner et al., 1969; Gittleman and Thompson, 1988).

In our study, we examined the factors that affect the swim speed of diving seals while manipulating three conditions (drag, buoyancy, and dive depth) and showed that only drag affected swim speed, which agreed with a previous theoretical prediction (Sato et al., 2010). We successfully confirmed this theoretical prediction using both qualitative and quantitative approaches. Calculating swim speeds using the maximum reported values of *C*_D_ suggested that the harness attachment tended to have greater hydrodynamic effects on the smaller animals (F97HA and F00BO) than it did on the larger animal – whereas the mechanism behind the greater reduction in swim speed between treatments C and B in larger animal remains unclear. Conceivably the larger animal had less *C*_D_ or a more hydrodynamic body than the smaller animals, but further study is required to confirm this suggestion given that Stelle et al. failed to find a similar relationship (Stelle et al., 2000).

Our study shows that swim speed can be used to estimate the energetic cost of a dive, and that diving animals swim at optimal speeds that minimize the energetic cost of diving and maximize the time that can be spent foraging at depth. Swim speeds can be obtained by knowing the metabolic and mechanical costs (Sato et al., 2010), which means that inverse calculations can be used to estimate energetic costs from swim speeds. We were unfortunately unable to measure the energetic cost for each dive because we allowed the sea lions to undertake bouts of dives, rather than single dives. However, further quantitative analyses of the amount of drag and energy cost for a single dive using respirometry would enable energy expenditures to be estimated using swim speed in breath-hold divers.
